# Three-Dimensional, High-Resolution Skeletal Kinematics of the Avian Wing and Shoulder during Ascending Flapping Flight and Uphill Flap-Running

**DOI:** 10.1371/journal.pone.0063982

**Published:** 2013-05-15

**Authors:** David B. Baier, Stephen M. Gatesy, Kenneth P. Dial

**Affiliations:** 1 Department of Biology, Providence College, Providence, Rhode Island, United States of America; 2 Department of Ecology and Evolutionary Biology, Brown University, Providence, Rhode Island, United States of America; 3 Division of Biological Sciences, University of Montana, Missoula, Montana, United States of America; College of the Holy Cross, United States of America

## Abstract

Past studies have shown that birds use their wings not only for flight, but also when ascending steep inclines. Uphill flap-running or wing-assisted incline running (WAIR) is used by both flight-incapable fledglings and flight-capable adults to retreat to an elevated refuge. Despite the broadly varying direction of travel during WAIR, level, and descending flight, recent studies have found that the basic wing path remains relatively invariant with reference to gravity. If so, joints undergo disparate motions to maintain a consistent wing path during those specific flapping modes. The underlying skeletal motions, however, are masked by feathers and skin. To improve our understanding of the form-functional relationship of the skeletal apparatus and joint morphology with a corresponding locomotor behavior, we used XROMM (X-ray Reconstruction of Moving Morphology) to quantify 3-D skeletal kinematics in chukars (*Alectoris chukar*) during WAIR (ascending with legs and wings) and ascending flight (AF, ascending with wings only) along comparable trajectories. Evidence here from the wing joints demonstrates that the glenohumeral joint controls the vast majority of wing movements. More distal joints are primarily involved in modifying wing shape. All bones are in relatively similar orientations at the top of upstroke during both behaviors, but then diverge through downstroke. Total excursion of the wing is much smaller during WAIR and the tip of the manus follows a more vertical path. The WAIR stroke appears “truncated” relative to ascending flight, primarily stemming from ca. 50% reduction in humeral depression. Additionally, the elbow and wrist exhibit reduced ranges of angular excursions during WAIR. The glenohumeral joint moves in a pattern congruent with being constrained by the acrocoracohumeral ligament. Finally, we found pronounced lateral bending of the furcula during the wingbeat cycle during ascending flight only, though the phasic pattern in chukars is opposite of that observed in starlings (*Sturnus vulgaris*).

## Introduction

Birds employ their wings for a broad array of locomotor tasks. Among these, uphill flap-running or wing-assisted incline running (WAIR) is of particular interest. During WAIR, the wings and legs are simultaneously engaged to scale steep inclines – a common behavior exhibited by juvenile as well as adult birds [1], [2]. WAIR and controlled flapping descent (CFD) are employed throughout development in all extant forms studied to date and have been suggested to be relevant to discussions of ecological survivorship of flightless young, as well as the evolutionary origin of avian flight [2]–[6].

Birds with intact flight feathers (remiges) can ascend steeper inclines during WAIR than those with clipped remiges [1], suggesting that the wings provide a climbing advantage. Measurements of body acceleration and substrate reaction forces demonstrate that wing flapping increases traction [7]. More recently digital particle image velocimetry (DPIV) of chukars confirmed and further refined this assessment, showing that aerodynamic forces, as opposed to inertial forces, were directly involved in generating the substrate-directed component of force [8]. Thus, compared to ascending flight at a similar trajectory, the wing path is expected to differ during WAIR in order to redirect the force towards the substrate, but by how much?

In a broad comparison of flapping behaviors, a consistent pattern of wing movement was found between WAIR at multiple inclines, level flight, and controlled-flapping descent across ontogeny. Using 3-D reconstruction from external wing landmarks, the sweep of the wing followed a narrow range of angles in reference to gravity, regardless of the body’s pitch or direction of travel [4], [5]. The relatively invariant stroke-plane angle (<20 degrees) and angle of attack in the global reference frame led to the hypothesis of a “fundamental” or “stereotypic” wing stroke, where the body is free to pitch through an arc of angles relative to the wing. This hypothesis predicts that the skeletal joints of the wing, particularly the glenohumeral joints, operate through a broad range of excursions during different behaviors to produce a similar global stroke-plane angle. Herein, we evaluate the forelimb skeletal kinematics during ca. 70 degree WAIR and ascending flight to better understand the underlying joint motions in light of predictions made from external video [4], [5].

The glenohumeral joint forms from the articulation of the bulbous, ovoid humeral head and the saddle-shaped glenoid supported by both the scapula and coracoid. A prominent acrocoracohumeral ligament (AHL) spans across the anterior surface of the glenoid from the elevated acrocoracoid process to the transverse sulcus on the humerus. Sy [9] implicated the AHL in restricting humeral pronation and, in part, controlling the pitch of the body between the wings. A recent force balance model suggests a more extensive role of the AHL as a critical element that stabilizes the glenohumeral joint [10], [11] to prevent dislocation by the primary flight musculature. The combination of glenohumeral morphology and AHL geometry are predicted to simultaneously constrain and control joint mobility but also permit a broad range of humeral paths during different behaviors. Hence, we hypothesize that the morphology of glenohumeral joint and AHL are consistent with the predicted shoulder movements of the stereotypic wing beat hypothesis [4], [5].

Many investigations of bird flight have measured external wing kinematics [12]–[22], but only a few existing studies record skeletal movements of bird flapping their wings [23]–[26], and these were at the time necessarily limited to single-plane fluoroscopy during level flight. Newly developed methods used here merge biplanar fluoroscopy with CT scan models to provide unprecedented accuracy and precision in reconstructing skeletal and joint movements of locomoting animals (XROMM, X-ray Reconstruction Of Moving Morphology) [25], [27], [28]. Dual X-ray has been used to study hummingbird wing mechanics [28], and although many aspects of WAIR have been studied, no data exist on the underlying skeletal movements. In this study, we explore the two extremes of wing function (flap running and ascending flight; Videos S1, S2, S3, S4) in adult chukars (*Alectoris chukar*) as animals ascend the same trajectory (ca. +70 degrees) in order to evaluate the underlying skeletal kinematics associated with these distinct flapping behaviors.

## Materials and Methods

### Animals

Two adult chukars were raised from hatchling at the University of Montana and transferred to the Brown Animal Care Facility. Animals were cared for and housed in accordance with an IACUC protocol that was reviewed and approved by the Brown University Institutional Animal Care and Use Committee. Brown University has an Animal Welfare Assurance (#A3284-01) on file with OLAW/NIH. Animals were trained to ascend to a refuge box both with and without a ramp present.

### General Methodology

We used markerless XROMM (X-ray Reconstruction of Moving Morphology) [25], [27] also known as Scientific Rotoscoping [25] to reconstruct skeletal motions of the forelimb bones. We combined biplanar X-ray video with digital skeletal models derived from CT scans of the same birds used in the video to reanimate the actual skeletal movements (Video S1). Six degrees of freedom (DOF) joint kinematics are then measured from these animated anatomical models. Complete descriptions of XROMM procedures are available at XROMM.org. Herein, we present only the salient details of the method relevant to the current study.

### Video Collection

**Figure 1:**
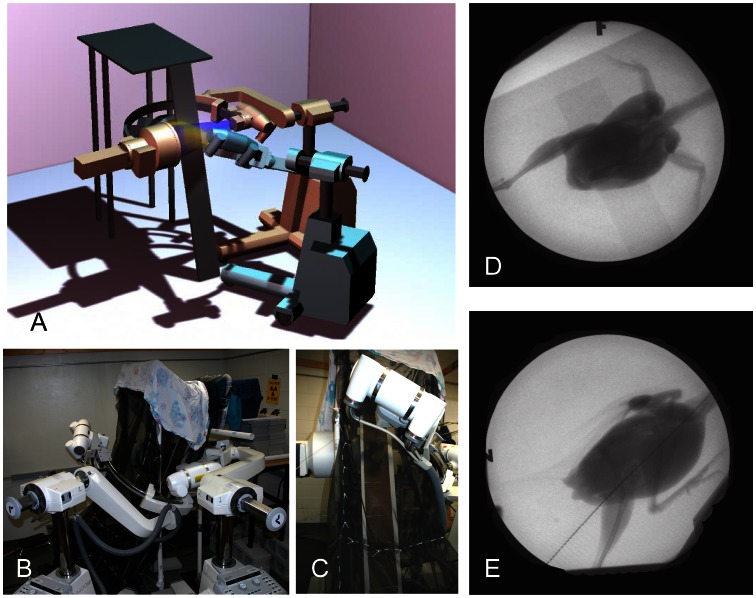
Experimental setup. A. 3-D model of the experimental setup showing the position of the imaging area of the two X-ray beams (yellow and blue cones). B. the actual setup. C. view from position of the animal handler (chukar on ramp between dual X-ray beams). D. dorsoventral X-ray view. E. lateral X-ray view. Note WAIR images from D and E have been mirrored horizontally.

The two C-arm X-ray machines were configured with a slightly oblique lateral view and a dorsoventral view (perpendicular to the ramp) (Fig. 1). A black nylon mesh enclosed the space around a 70 degree, 1.75 m ramp, which spanned between the floor and an elevated perch box and passed through the overlapping beam field. Two synchronized, high-speed Photron 1024×1024 cameras with shutter speeds of 1/6000s captured digital video of the image intensifiers’ video output windows at 500 fps. Although cameras were mounted at the output windows of the image intensifiers, mirroring each video left to right provided a view as seen from the X-ray emitter (source) rather than the detector for XROMM analysis. Eight trials with at least one complete wingbeat were chosen for full analysis from a total of 82 trials (2 trials of WAIR and 2 trials of ascending flight per bird). On average, birds were exposed to the X-ray beams 0.36 seconds per trial. Runs with deviations from the desired behaviors (e.g., stopping on the ramp during WAIR or paddling feet towards the netting in ascending flight) were discarded, as were runs lacking complete view of all elements of the wing and shoulder girdle for at least one full wingbeat.

### Calibration

We removed image distortion by capturing an image of a hexagonally perforated metal grid placed directly on the image intensifier. A transformation matrix was derived using Matlab (Mathworks; version 2011b) with a local weighted means solver to correct the distorted image back to a regular grid from each camera [27]. Then, an image of an acrylic calibration object with 3 mm stainless steel balls spaced 65 mm apart in the XYZ direction was used to calibrate the 3-D space. Direct linear transformation (DLT) coefficients were derived for each camera [27], [29].

### Ct Scans and Bone Models

**Figure 2:**
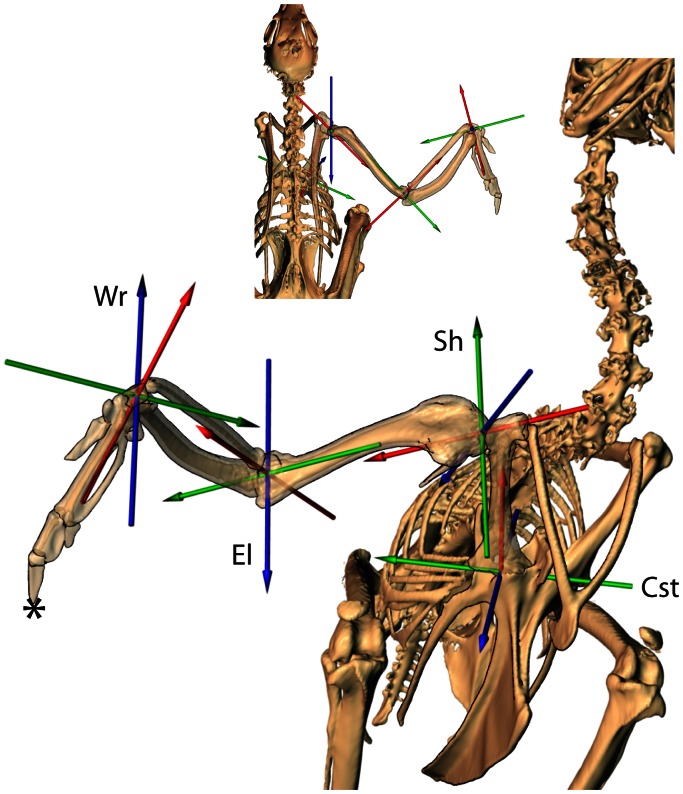
Joint coordinate system overview. Each joint is allowed 6 degrees of freedom (DOF) about an established coordinate system based on inertial axes of the downstream bone (e.g. humeral inertial axes determine glenohumeral axes). Joint rotations are ordered such that rotation about the blue axis moves the other two axes and bone model; rotation about green moves only the red axis and bone model; and rotation about the red axis only affects the bone model. Rotation order follows blue, green and red in each joint depicted. Cst = coracosternal joint: blue – abduction/adduction; green – protraction/retraction; red – long axis rotation. Sh = shoulder (glenohumeral), El = elbow, and Wr = wrist joints: blue – elevation/depression; green – protraction/retraction; red – pronation/supination. Note, none of the joints are depicted in their zero reference pose. For example, the elbow is extended 120 degrees from its zero position. Specifics for each joint are discussed in results. The * marks the distal-most point on the *os phalanx distalis digiti* used to determine upstroke and downstroke transitions.

The animals were euthanized and frozen following data collection. CT scans were collected at Rhode Island Hospital (technique; 80 kVp, 400 mA, 0.625 mm slice thickness). Individual bone models were segmented and saved as polygonal meshes using Amira 4.0 (Mercury) (Fig. 2). To provide a consistent frame of reference between individuals, we calculated the center of mass and inertial axes for each bone model by treating it as a solid [30] using Matlab. These inertial axes, combined with anatomical landmarks when needed, were used to create an anatomical reference pose and a hierarchical digital “puppet” (Fig. 2) in Maya 2010 (Autodesk) [25]. Potential errors in the estimation of inertial axes are variable for each bone and for axes within bones. For example, the sternum inertial axis representing the long axis was consistent between our two specimens. However, the other two axes were offset relative to each other. Hence, we used the long axis measured from inertial axes but set the other two axes based on anatomical landmarks that were clearly visible and representative of sturdy parts of the bone (articular facets for the coracoid, rostral process of the sternum). The inertial axes of the coracoids, humerus and ulna were visually assessed to be at least as good as using the less repeatable method of picking anatomical landmarks. Any method of attempting to establish consistent axes between two or more individuals is subject to “kinematic cross-talk” [31] where some of the rotation about one axis is interpreted within another axis. This is a major issue when attempting to estimate bone/joint systems from external markers but is much less problematic when directly visualizing the skeleton as in our study. Joint coordinate systems (JCS) [32] were established for each joint to measure translations and rotations. Rotation order was xyz in Maya which means that rotations first occur about z, then y, and x last when moving from the reference pose to the animated pose (Blue, Green, Red; Fig. 2). Specific criteria for determining the reference pose of each joint and are discussed in results.

### Animation

In markerless XROMM, 3-D polygonal bone models are manually positioned and oriented in Maya to match the X-ray shadow of the bone simultaneously in both camera views. Each joint is allowed six degrees of freedom and measured using a consistent joint coordinate system [32]. Since the model is hierarchical, the most upstream elements (pelvis and sternum) are aligned to the entire video sequence, followed by the downstream elements in order: coracoids, humerus, forearm and hand. Ideally, the proximal element is perfectly aligned on the first pass, but occasionally, attempts to match downstream elements reveal inconsistencies in upstream positioning (by unlikely movements or relative joint positions). Hence, segments of the animation are reworked iteratively to refine the animation. The first attempt to position and orient the coracoids is done assuming symmetrical movements. Additional passes refine each individual coracoid to “fine-tune” the shoulder girdle movements.

### Data Processing and Statistics

#### Wingbeat timing

A few measures, such as average velocity of the body and relative time spent in downstroke and upstroke, were taken from raw timing. However, in order to compare joint movements for different behaviors and individuals, we divided wingbeats into downstroke and upstroke phases. Kinematic data were time-scaled to percent phase (downstroke: 0% = top of upstroke to 100% = bottom of downstroke; upstroke: 0% = bottom of downstroke to 100% = top of upstroke). Average wingbeats were determined by taking the average of each DOF at each percentage timestep of upstroke and downstroke for the 4 trials of each behavior (Video S5). Although studies using standard light video generally rely on a primary feather tip or the wrist position to define phase transitions e.g. [22], we chose to define upstroke/downstroke by tracking the distal-most point on the *os phalanx distalis digiti* (Fig. 2) relative to the vertebral column (inertial axes of notarium). Using the fingertip let us account for the combined contributions of shoulder, elbow and wrist movements and to compare peak elevations of each segment for all wingbeat cycles.

We discovered that for some wingbeats, the maximum elevation and depression of the humerus were offset from the fingertip turnaround. For example, the shoulder may begin elevation while the fingertip is still moving downwards. This led us to compare peak elevation/depression for distal points on the humerus, ulna, and fingertip for all wingbeats.

All other calculations were done in Matlab. Displayed means and standard deviations were calculated by taking the time-step average of all wingbeats per behavior across all birds. To test for statistical differences between behaviors, and to account for the non-independence of multiple measures of the same bird, we present statistical tests from repeated measures ANOVA. Given the small attainable sample size, only a few variables differ significantly. However, we also highlight several variables that are suggestive of differences but would require more samples to demonstrate statistical significance. Individual degrees of freedom are treated as independent variables statistically. However, it should be noted that ordered rotations are not truly independent.

#### Validation

Manual model registration (rotoscoping) accuracy is affected by a variety of sources [25], including distortion errors in the video images, calibration errors, bone model reconstruction, X-ray opacity and morphological distinctness of individual bones, and overlapping X-ray shadows from multiple bones. To gauge our accuracy, we compared marker-based and markerless results for the same sequence of video. We implanted 3–4 steel beads (1 mm diameter) in each of the sternum, coracoid, and humerus of a chukar carcass and collected dual X-ray video while manipulating the wing with a dowel attached to the manus. First, bones were animated using marker-based methods [27] as the “gold standard” for bone tracking [33]. Second, marker shadows were removed from all X-ray frames (Photoshop CS5, Adobe Systems Inc.; stamp tool), and bone movements were reconstructed by rotoscoping the same sequence of video [34]. We calculated mean absolute error as the average absolute value of the differences between marker-based and rotoscoped bones using the joint coordinate systems established for the coracosternal and glenohumeral joints.

Individual degrees of freedom may be misleading because a translational error may be compensated for by rotational correction or vice-versa. For example, if the proximal end of the coracoid is misplaced 1 mm to the right, an additional rotation about the proximal pivot could move the distal end of the bone to nearly the correct position. In this case, the translation and compensating rotation would both be magnified as errors when looking at the 6 DOF of the coracosternal joint, but the coordinates of points on the distal coracoid would be quite accurate. Therefore, we also calculated mean absolute error for the distance between the same point on the distal coracoid and humerus for rotoscoped and marker-driven animations. We assume the marker-driven as the gold standard, but it should be noted that marker-driven estimates also have error [27].

## Results

### Wingbeat Timing

The elbow and wrist moved upward prior to the tip of the manus at the downstroke/upstroke transition during ascending flight (AF: elbow:−4.0% ±2.1, wrist: −2.0% ±2.2) and Wing-Assisted Incline Running (WAIR: −6.3% ±3.0, −3.1% ±4.5). The upstroke/downstroke transition during AF showed almost no timing offset relative to the tip of the manus (elbow 0.5% ±1.2, wrist 0.4% ±1.1), but during WAIR the elbow (−2.0% ±4.3, wrist −3.1% ±3.0) offsets were both larger in magnitude and more variable in pattern.

### General Body Kinematics

**Figure 3:**
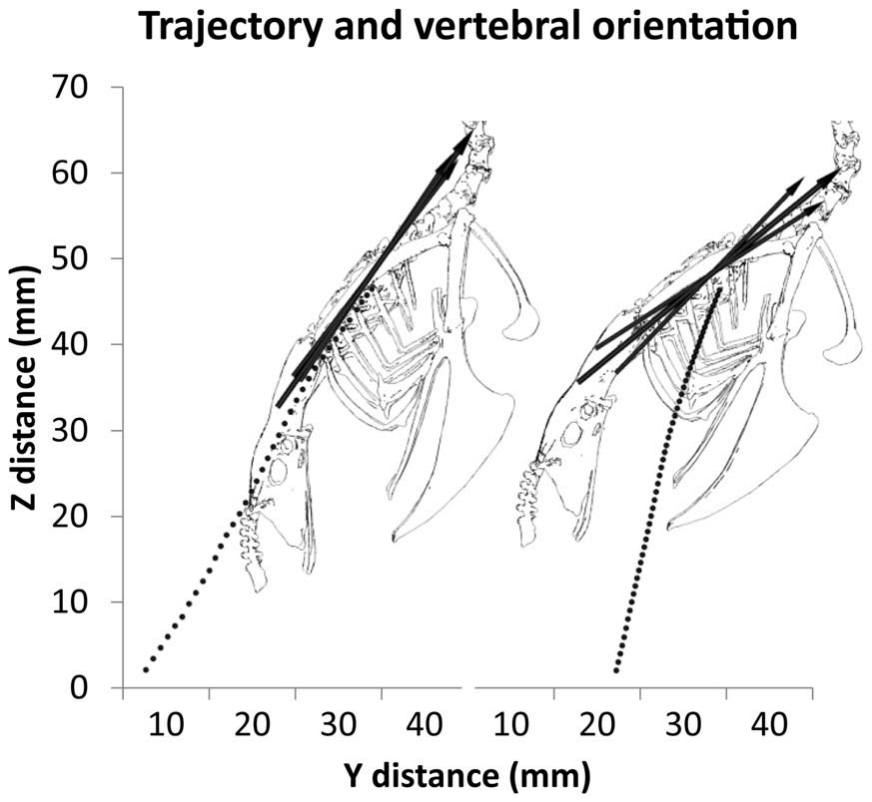
Body pitch and trajectory. Average trajectory of the body (dotted line) compared to the vertebral axis orientation between ascending flight (AF; n = 4) and WAIR (WAIR; n = 4). The larger middle arrow indicates the mean vertebral pitch, the two smaller arrows are the average maximum and minimum pitch angles for the respective behavior. Note that pitch is less steep, more variable and differs from the trajectory line in WAIR compared to AF. Also, AF is nearly twice as fast as WAIR as indicated by the space between points sampled every 20 ms.

We measured body velocity and trajectory relative to horizontal by tracking the 3-D position and orientation of the *spina interna rostri* of the sternum as a proxy for the center of mass [9]. On average, AF was faster than WAIR (AF: 2.0±0.12 m/s; WAIR: 1.2±0.22 m/s) although not statistically different (Table 1). In addition, the trajectory of ascent relative to horizontal (Fig. 3) was steeper during WAIR (AF: 59.8 degrees ±8.4; WAIR: 74.6±3.9) despite the fact that the starting position and ending perch were the same in both. The body axis (long axis of the notarium) was more steeply pitched during AF (53.4 degrees ±4.0; and WAIR: 39.6 degrees ±4.4) and fluctuated less as indicated by a smaller range in pitch (AF: 4.1±1.3; WAIR 13.3 degrees ±4.4). Roll and yaw did not differ between behaviors and were of small magnitude (mean range: roll, 7.9 degrees ±3.6, p-value 0.490; yaw, 7.4 degrees ±4.0, p-value 0.477).

**Table 1:** Summary of body velocity, trajectory, orientation and wing movement relative to the global coordinate system.

Summary of body movement in global coordinate system					
	AF		WAIR		p-value
average velocity (m/s)*	2.0	±0.12	1.2	±0.22	0.184
trajectory (degrees)*	59.8	±8.4	74.6	±3.9	0.195
pitch(degrees)*	53.4	±4	39.6	±4.4	0.199
pitch range (degrees)*	4.1	±1.3	13.3	±4.4	0.118
roll range (degrees)	8.4	±4.2	7.4	±3.5	0.490
yaw range (degrees)	5.9	±2.4	9.0	±2.4	0.477
fingertip path (degrees)	**138.5**	±**4.6**	**106.5**	**±3.7**	**0.044**

Bold = significant difference based on repeated measures ANOVA.

* = non-significant but suggest possible differences.

### Coracosternal Joint and Furcula

**Figure 4:**
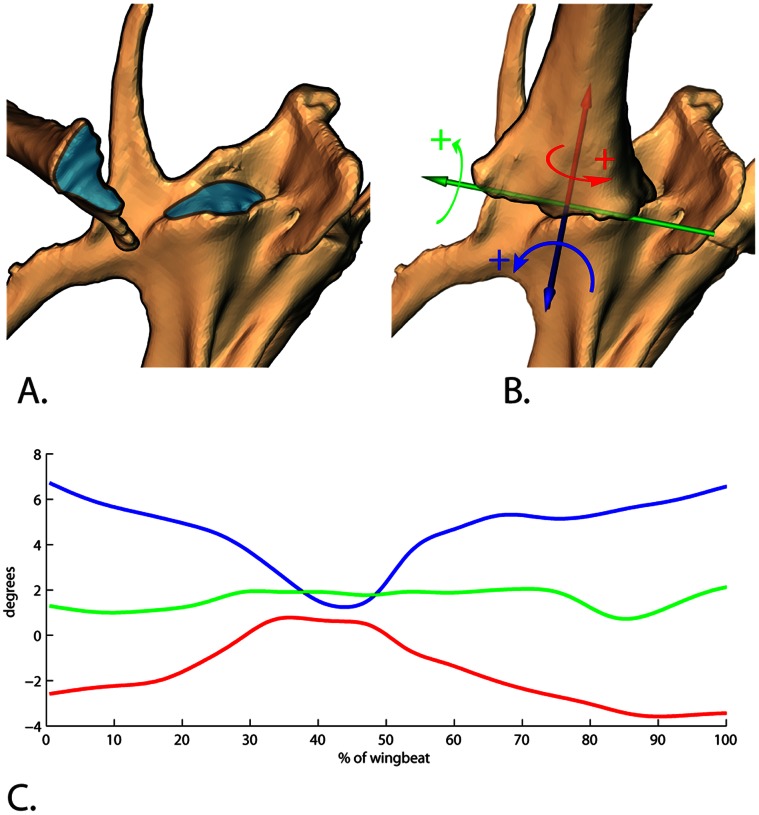
Coracosternal articulation and coordinate system. A. the articular surface (blue) of the proximal coracoid fits into the elongate sulcus (blue) on the anterior end of the sternum. B. the joint coordinate system used to measure coracosternal movements. Blue = abduction/adduction axis; Green = protraction/retraction; Red = long axis rotation. C. rotation at this joint was primarily found during AF. The majority of movement during downstroke was adduction, causing medial movement of the distal coracoid. Almost no protraction/retraction rotation was indicated, but internal long axis rotation was found in downstroke. However, it should be noted that the amount of rotation in all cases is within the range of error for individual degrees of freedom (Table 3). Our validations show that while the placement of the distal coracoids is highly reliable, the actual movements at the coracosternal joints are less accurate given that measures are compounded from both sternal and coracoid registration during rotoscoping.

The coracosternal joint is formed by the flattened *facies articularis sternalis* of the coracoid and the *sulcus articularis coracoideus* of the sternum (Fig. 4a). The articular facet occupies the medial 70% of the proximal coracoid (12.3 mm) in our adult chukars, with the expanded lateral process comprising the remaining width. On the sternum, the coracoid sulcus faces anteriorly at its medial margin and anterolaterally at its lateral margin. When looking from the frontal view with the vertebral axis being horizontal, the shafts of the coracoids project laterally ∼65 degrees. From the lateral view, they project anteriorly ∼27 degrees from the coracosternal joint (based on CT scans). Because the distal coracoids are joined by the crura of the furcula, movement at the coracosternal joint results in furcular bending. Therefore, we measured coracosternal movements in conjunction with furcular spread (distance between the distal coracoids).

We defined the coracosternal joint coordinate system (JCS) using the inertial axes of the coracoid (Fig. 4b); abduction/adduction spins about the axis of greatest inertia (blue in Figs. 2 and 4b), long axis rotation happens about the axis of least inertia (red in Figs. 2 and 4b) and protraction/retraction occurs around an axis perpendicular to the other two (green in Figs. 2 and 4b). The rotation order follows: 1) abduction/adduction, 2) protraction/retraction, and then 3) long axis rotation (blue, green, red; Fig. 4). The blue axis remained fixed to the sternum; the red axis was fixed to the coracoid and the green axis “floats” to stay perpendicular to both [32].

The majority of coracosternal motion underlying the furcular spread during AF resulted from abduction/adduction (5.6±1.6 degrees; blue axis in Fig. 4b, c) and long axis rotation (4.4±0.9 degrees; red axis in Fig. 4b, c). Protraction/retraction was 1.4±1.1 degrees. As the coracoids adduct during downstroke, they rotate about their long axis such that the laterally-facing surface faces more anteriorly.

**Figure 5:**
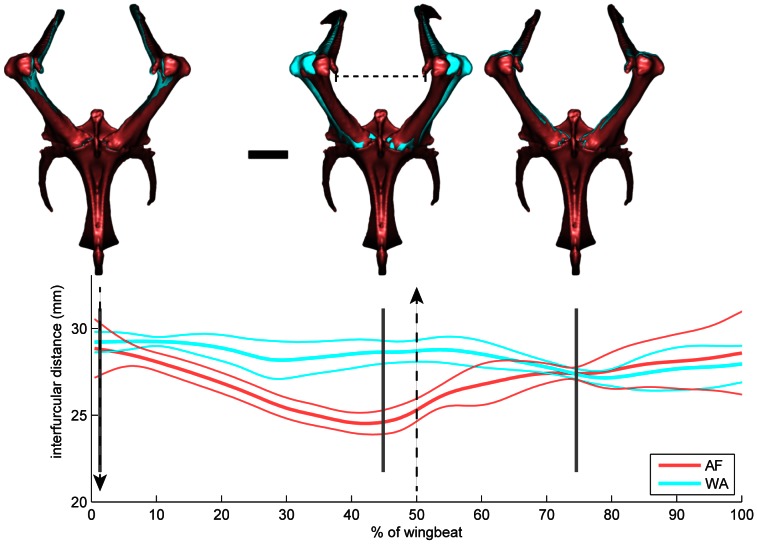
Furcular deformation. Interfurcular distance change comparing AF (red) and WAIR (blue) wingbeats. During both behaviors, the interfurcular distance expands to 129% of resting length near the upstroke/downstroke transition. During WAIR the interfurcular distance remains relatively unchanged, but during AF the coracoids adduct towards the resting configuration during downstroke and then re-expand during upstroke. Dashed vertical lines indicate beginning of downstroke (downward arrow) and beginning of upstroke (upward arrow). Gray vertical lines indicate frames of video imaged above (1, 44, 74). Horizontal dashed bar in middle image is resting length. Scale bar = 10 mm.

AF produced greater coracosternal motion and associated furcular spread range (Fig. 5; Videos S6, S7; AF: 5.0±0.07 mm; WAIR: 2.5±0.04 mm). Furcular spread during flapping motion was always greater than resting distance (23.0 mm). During both behaviors, the furcula spread to a maximum of ca. 129% of resting distance, but the furcula only recoiled to near resting distance during AF. During WAIR, the furcula stayed spread between 129 and 118% of resting distance for the entire wingstroke. During AF, mean maximum spread distance (29.6 mm) was reached at the upstroke/downstroke transition. Mean minimum distance (24.6 mm) occurred at 80% of downstroke (Fig. 5). During WAIR, unlike AF, the interfurcular distance decreased slightly and then expanded during upstroke reaching the minimum at 60% of upstroke. A smaller decrease occurred during downstroke (Fig. 5).

### Glenohumeral Joint

The glenoid, supported by bony facets on the omal ends of both the coracoid and scapula, articulates with the ovoid humeral head. The JCS was oriented by positioning the humeral inertial axes at the center of a sphere approximating the shape of the humeral head (Fig. 2). The zero pose was defined relative to the vertebral reference frame with the long axis of the humerus pointing directly laterally (perpendicular to the glenoid), the elevation/depression axis running parallel to the vertebral column, and the protraction/retraction axis oriented dorsoventrally. In this pose, the deltopectoral crest points anteriorly and the long axes of the distal condyles and humeral head orient dorsoventrally. The rotation order of the JCS was as follows: 1) elevation/depression, 2) protraction/retraction, and then 3) pronation/supination (blue, green, red; Fig. 2). The e/d axis (blue in Fig. 2) remained fixed to the glenoid, the p/s axis (red in Fig. 2) was fixed to the humerus and the p/r axis (green in Fig. 2) “floated” to remain perpendicular to both [32].

**Figure 6:**
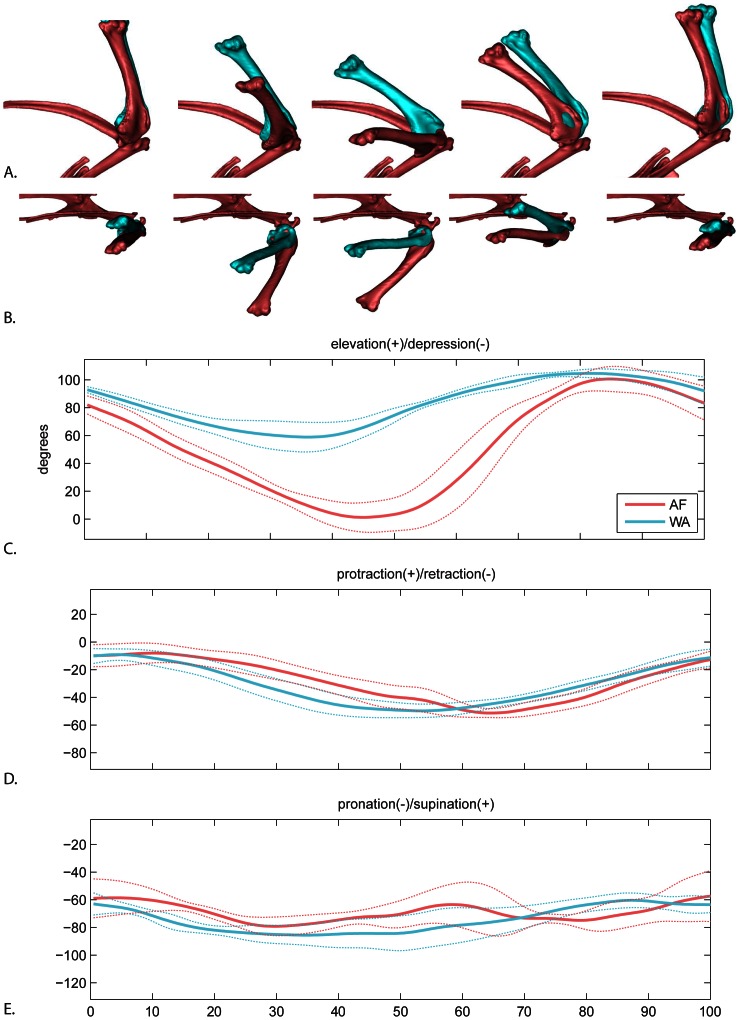
Glenohumeral motion. A. lateral view and B. dorsal view of average AF (red) and WAIR (blue) wingbeats at 0, 25, 50, 75 and 100% of the cycle. Humerus shown relative to a fixed shoulder girdle. C–E mean and standard deviations for each rotational degree of freedom. Time scale 0 to 50% = downstroke, 50–100% = upstroke.

Humeral elevation/depression showed the greatest difference between AF and WAIR for any degree of freedom in this study (Fig. 6C). In terms of timing, the humerus depresses to its minimum and begins elevating prior to the end of downstroke for both behaviors. However, peak depression is reached earlier in the downstroke in WAIR (WAIR: 73.8% ±4.6 downstroke; AF 92.0% ±9.3 downstroke). During upstroke, humeral timing was similar between AF and WAIR, reaching a combined peak at 67.6±10.5% upstroke and then beginning to depress prior to the wingtip reaching its highest point.

In terms of magnitude, total range of humeral elevation/depression was greater during AF (101.6±5.5 degrees) than WAIR (47.6±11.02) although not significantly different (p-value 0.063). Mean peak elevation was slightly higher in WAIR (105.9±2.1 degrees) compared to AF (101.6±8.7 degrees). Average maximum depression revealed the largest difference between behaviors. The humerus approached horizontal relative to the coronal plane in AF (−0.4±12.3 degrees) but only reached 58.2±9.8 degrees above this plane during WAIR (Fig. 6; Table 2).

**Table 2:** Summary of individual rotational degrees of freedom for each joint comparing AF (ascending flight) and WAIR (Wing-Assisted Incline Running).

Summary comparison of AF and WAIR individual DOF											
		Range (mean)	std	p	Max (mean)	std	p	Min (mean)	std	p	
Glenohumeral											
elevation/depression	AF	101.6*	5.4	0.063*	101.1	8.7	0.463	−0.4*	12.3	0.103*	
	WA	47.6*	11.0		105.9	2.1		58.2*	9.8		
protraction/retraction	AF	45.5	5.9	0.516	−6.8	5.4	0.336	−52.3	3.0	0.617	
	WA	42.1	6.5		−8.4	4.0		−50.5	5.0		
pronation/supination	AF	35.8	15.1	0.630	−48.8	14.3	0.528	−84.6	6.4	0.284	
	WA	30.6	9.8		−57.8	2.7		−88.3	9.6		
elbow											
flexion/extension	AF	91.3*	5.2	0.205*	126.7	4.4	0.176	35.4*	1.8	0.130*	
	WA	60.8*	9.6		121.7	5.0		60.9*	9.4		
abduction/adduction	AF	**47.0**	9.4	**0.041**	44.1*	16.7	0.071*	−2.9	9.5	0.272	
	WA	**28.4**	3.6		15.9*	6.6		−12.5	4.9		
long axis rotation	AF	37.7	8.5	0.249	19.3	13.7	0.496	−18.4	8.5	0.225	
	WA	29.1	7.3		9.0	10.0		−20.1	6.6		
wrist											
flexion/extension	AF	90.0*	17.8	0.243*	147.9	15.5	0.034	57.9	24.2	0.524	
	WA	48.2*	15.4		120.4	17.0		72.2	10.7		
abduction/adduction	AF	79.4*	5.4	0.076*	9.5	3.1	0.946	−69.9*	5.1	0.109*	
	WA	50.2*	4.4		8.8	8.3		−41.4*	5.8		
long axis rotation	AF	48.1	13.0	0.262	6.5	15.2	0.166	−41.6	4.6	0.685	
	WA	33.4	12.6		−3.3	14.5		−36.8	15.9		

Bold = significant difference based on repeated measures ANOVA.

* = non-significant but suggest possible differences.

During both AF and WAIR, the humerus primarily retracts during downstroke and protracts during upstroke (Fig. 6D). However, timing varies between behaviors. Retraction ends at the downstroke/upstroke transition during AF but continues into upstroke during WAIR.

The humerus primarily pronates during downstroke and supinates during upstroke (Fig. 6E). The timing of beginning supination is relatively similar, occurring approximately midway through downstroke (WAIR: 25.3% ±17.1 wingstroke; AF 29.4% ±3.8). Although both behaviors were of similar total magnitude (AF: range 35.8±15.1, max −48.8±14.3, min −84.6±3.0; WAIR: range 30.6±6.5, max −57.8±2.7, min −88.3±9.6), during AF, the humerus underwent a second pronation phase during upstroke prior to fully supinating.

### Elbow

The elbow joint forms between the distal humerus and proximal ulna and radius. We did not attempt to quantify movement between the radius and ulna in this study, although relative motion is likely [35]. To place the JCS, we visually fit a sphere to the distal condyles of the humerus to mark the position of the axes. The orientation of the axes matched the inertial axes of the ulna. Flexion/extension occurs about an axis passing through the distal condyles of the humerus (blue in Fig. 2), long axis rotation follows the axis of least inertia (red in Fig. 2), and abduction/adduction (green in Fig. 2) remains perpendicular to both. In the zero position, the ulna is flexed parallel to the humerus.

**Figure 7:**
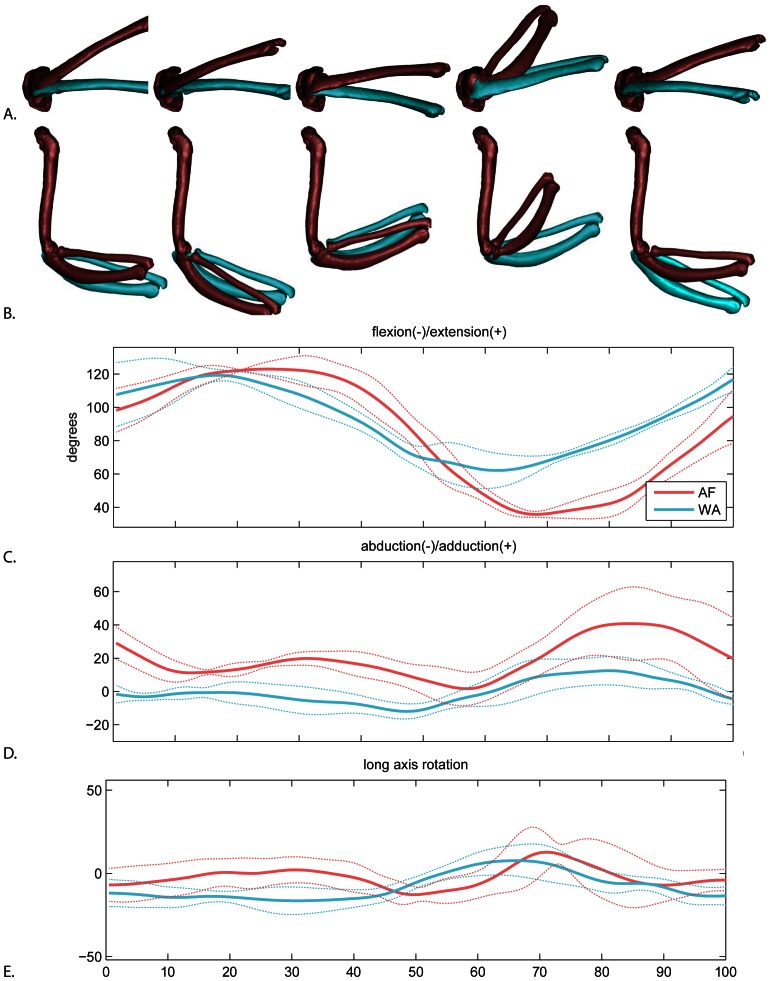
Elbow motion. A. lateral view and B. dorsal view of average AF (red) and WAIR (blue) wingbeats at 0, 25, 50, 75 and 100% of the cycle. Ulna and radius shown relative to a fixed humerus. C–E. mean and standard deviations for each rotational degree of freedom. Time scale 0 to 50% = downstroke, 50–100% = upstroke.

Non-significant but suggestive differences between WAIR and AF occur in both timing and magnitude of flexion/extension (Fig. 7C) and magnitude of abduction/adduction (Fig. 7D; Table 2). During early downstroke of both behaviors, the ulna re-extends from the previous stroke cycle. Peak extension is reached earlier in WAIR (21±11% downstroke; AF 46±15%). During the remaining downstroke, the elbow flexes. Peak flexion is also earlier in WAIR (28±10% upstroke; AF 45±9%). AF produces a higher range of flexion/extension. A similar maximum extension is reached in both behaviors, but the AF elbow reaches narrower angle of flexion (AF: range 91.2±5.2, max 126.7±4.4, min 35.4±1.8; WAIR: range 60.8±9.6, max 121.7±4.0, min 60.8±9.4).

Abduction range is significantly greater during AF (47.0±9.4 compared to WAIR: 28.4±3.6) with the elbow remaining ca. 15 degrees more abducted over the entire wingbeat during AF. The greatest abduction difference occurs in late upstroke (Fig. 7D). Long axis rotation showed no clear pattern (Fig. 7E).

### Wrist

**Figure 8:**
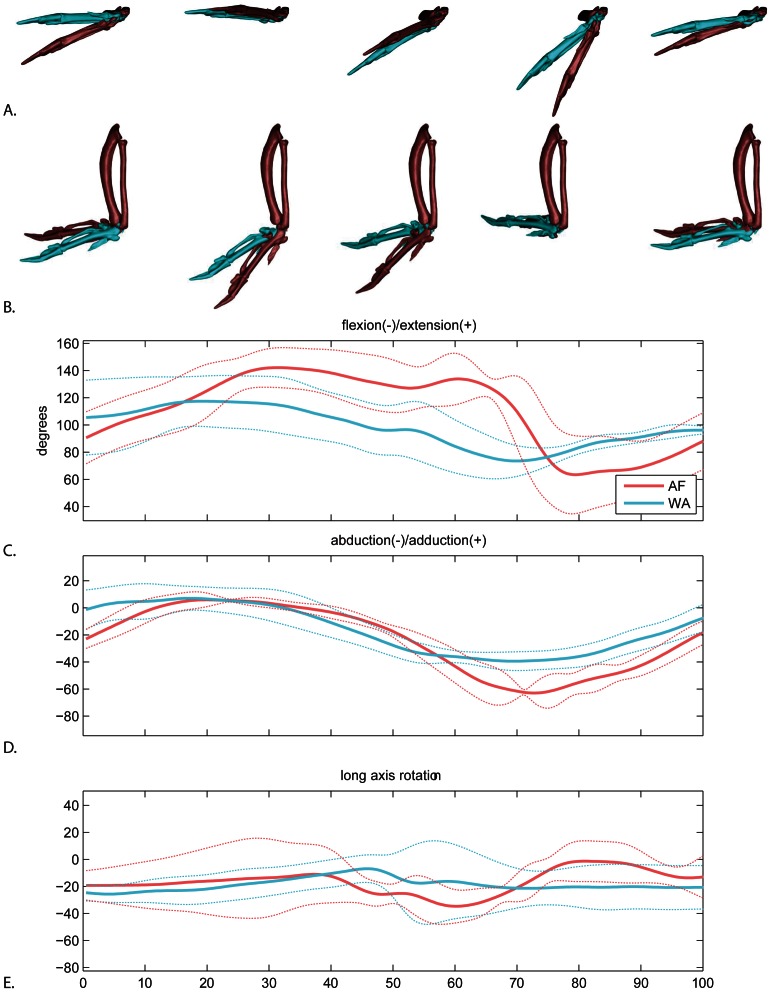
Wrist motion. A. lateral view and B. dorsal view (below) of the average AF (red) and WAIR (blue) wingbeats at 0, 25, 50, 75 and 100% of the cycle. Manus shown relative to a fixed antebrachium. C–E. mean and standard deviations for each rotational degree of freedom. Time scale 0 to 50% = downstroke, 50–100% = upstroke.

Here we define wrist motion as the orientation of the metacarpus relative to the ulna. As with the other joints, the more distal element’s inertial axis (the metacarpus) is used to establish the orientation of the JCS (Fig. 8). The primary axis is flexion/extension (blue axis in Fig. 2), followed by ad/abduction (green axis in Fig. 2) and long axis rotation (red axis in Fig. 2). The position was established by placing a sphere in the space between the metacarpus and ulna. In zero pose, the metacarpus is folded back parallel to and in the plane of the ulna, such that when looking from dorsal view, wrist extension produces movement in the same plane as elbow extension.

The largest difference in wrist motion between AF and WAIR was total magnitude of flexion/extension (Fig. 8C; Range: AF 90±17.8; WAIR 48.2±15.4). The wrist extended to a much greater degree during downstroke in AF and maintained the extension well into upstroke, whereas the wrist began flexing much earlier in the wingbeat during downstroke of WAIR (as with the elbow). Abduction (angled dorsally relative to the horizontal plane of the ulna) was minor in both behaviors; the majority of movement was adduction (Fig. 8D; AF 69.9±5.1; WAIR 41.4±5.8). Both behaviors followed a similar timing pattern of being near horizontal during downstroke then gradually adducting during late downstroke. The primary difference occurred in mid-upstroke when the wrist adducted to a greater degree during AF. Long axis rotation of the wrist was particularly noisy with no distinctive pattern (Fig. 8E).

### Validation

Here, we treat marker-driven reconstructions as the gold standard for comparison with the rotoscoped bones. The mean standard deviations for within bone inter-marker distances in the marker driven reconstructions were 0.08 mm, which is consistent with previous measures of the precision for this XROMM system [27]. For the sternum, coracoid and humerus, the mean difference between marker-driven and rotoscoped motion varied by degree of freedom, with long axis rotation producing the highest degree of error all bones (Table 3).

**Table 3:** Translation and rotation residuals (mean followed by standard deviation in parentheses) at joint pivots comparing rotoscoped bones to marker driven bones.

Validation test residuals at joint pivot							
	Bone length (mm)	Tx (std)	Ty (std)	Tz (std)	Rx (std)	Ry (std)	Rz (std)
sternum	84.5	0.57 (0.50)	0.76 (0.34)	0.46 (0.39)	−4.62 (2.49)	−0.26 (0.54)	2.62 (0.79)
coracoid	42.7	0.80 (0.50)	−0.25 (0.33)	0.26 (0.16)	3.84 (2.93)	−0.29 (0.68)	−3.50 (1.31)
humerus	57.2	−0.24 (0.49)	−0.37 (0.47)	0.24 (0.25)	−1.10 (3.80)	1.95 (1.34)	−1.21 (2.17)

Translations are in mm and rotations are in degrees. Bone lengths are the maximum length.

Individual degrees of freedom at a joint are not independent. Errors in translation may be compensated for by adjusting a rotational degree of freedom or vice versa (see Materials and Methods). Hence, we analyzed error by comparing both measured translational and rotational degrees of freedom at the joint (Table 3) and by comparing residuals of points at the distal ends of the two bones (Table 4).

**Table 4:** Validation residuals at distal points comparing rotoscoped bones to marker driven bones.

Validation test residuals distal point on bone					
	Bone length (mm)	X (mm)	Y (mm)	Z (mm)	
sternum	84.5	1.87 (0.69)	1.33 (0.41)	1.09 (0.21)	
coracoid	42.7	−0.11 (0.32)	0.59 (0.27)	0.32 (0.39)	
humerus	57.2	0.43 (0.36)	0.15 (1.05)	0.00 (0.37)	

Joint translational differences for all rotoscoped bones were between 0.25 and 0.8 mm. Rotational differences were higher for the sternum and coracoid, in particular Rz (axis fixed to the proximal bone) and Rx (axis fixed to the distal bone), which ranged between ca. 3 and 5 degrees, while Ry retained much lower differences (ca. 0.25). Ry is the pitching axis for the sternum and the humeral protraction/retraction axis, both of which are clearly determined from the lateral view X-ray. It seems likely that that the errors in the other two axes were linked compensations – an offset in one required an adjustment in the other. Humeral rotational errors were consistently lower about all three axes (ca. 1.5 degrees).

Coordinate offsets of the distal point varied between bones, with the sternum having the highest residuals (1.4±0.4 mm), followed by the coracoid (0.3±0.3 mm) and humerus (0.2±0.6 mm). Coracosternal joint motion is therefore the weakest measure and glenohumeral joint motion the strongest. The coracosternal joint movement accuracy is limited by placement of both the sternum and coracoid. However, the placement of the distal coracoid is still quite accurate. Thus, joint motions can compound errors in rotational and translational degrees of freedom but still yield accurate placement of the distal coracoid. This also suggests that furcular spread measures are within the range of accuracy and that glenohumeral translations are not impacted by the lower resolution of sternal and coracoid alignment.

### Joint Contributions to Wing Path

**Figure 9:**
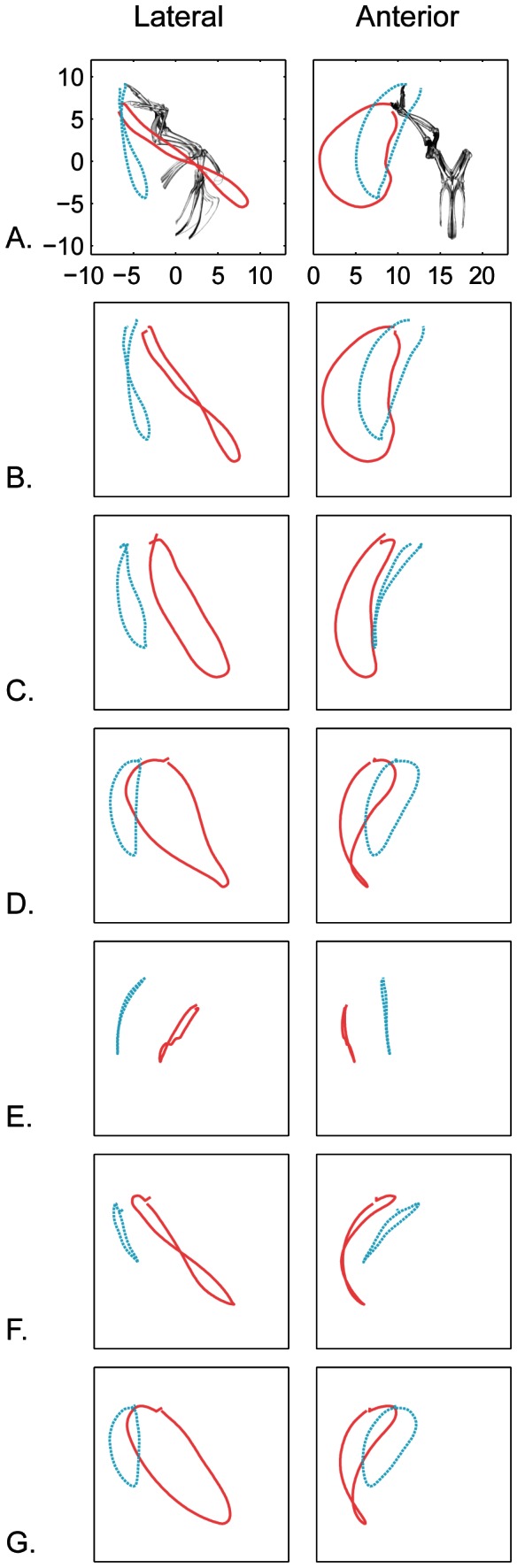
Joint contributions to fingertip path. A. wingtip path relative to the glenoid in a gravitational reference frame in lateral (left column) and anterior (right column) views for AF (red) and WAIR (blue). B – G show the paths after sequentially fixing one or more joints in their mean orientation and position throughout the complete wingbeat. B. pitch frozen. C. pitch and wrist motion frozen. D. pitch, wrist, and elbow motion frozen. E–G show the path after removal of each of the three degrees of freedom independently from glenohumeral motion. E. elevation/depression removed. F. protraction/retraction removed G. pronation/supination removed.

We measured fingertip path at the distal-most vertex on *the phalanx digiti majoris* (Fig. 9). When viewed laterally in a gravitational (fixed body translations but not rotations) reference frame, the fingertip path is more steeply angled (mean 101.6 degrees) during WAIR, and less steep (mean 142.8) during AF (Fig. 9A). Differing pitch of the vertebral axis accounts for 14 degrees of the 41 degree offset between behaviors (Fig. 3). Sequential removal of the effects of wrist and elbow motion does not eliminate the difference in path angle. While keeping the elbow and wrist fixed, removal of each rotational degree of freedom from the glenohumeral joint independently shows that elevation/depression is most responsible for the difference in wingtip path angle (Fig. 9).

We also assessed individual joint contributions to fingertip path by calculating the percent change in gravitational (inertial) X,Y and Z position of the fingertip (wrist, elbow joint, glenohumeral; Fig. 9, Tables 5, 6). Not surprisingly, the glenohumeral joint accounts for the majority of fingertip movement in each of the vertical, fore-aft, and mediolateral directions for both AF and WAIR (Table 5). Although glenohumeral motion dominates, wrist and elbow account for a slightly larger proportion of mediolateral displacement (particularly in AF) compared to vertical and fore-aft in which wrist and elbow oppose each other (a negative number in Table 5 indicates an increase in movement of the fingertip when the joint is frozen). For example, during both AF and WAIR, the elbow and wrist oppose each other in the fore/aft plane, but during AF, the wrist has a greater relative impact.

**Table 5:** Percent change in fingertip motion after removing contributions from subsequent distal-most joint.

Joint contribution to fingertip movement				
Joint		Vertical% Δ	Fore/aft% Δ	Mediolateral% Δ
**Wrist**	AF	−15.6	13.0	23.5
	WAIR	9.7	−16.0	25.6
**Elbow**	AF	16.2	−10.7	5.0
	WAIR	7.4	21.2	−7.7
**Glenohumeral**	AF	89.7	91.4	70.2
	WAIR	82.6	90.3	81.0
**Coracosternal and body pitch**	AF	9.8	6.3	1.4
	WAIR	0.3	4.5	1.1

Joints are frozen at their average position and orientation through the mean complete wingbeat. Contribution is based minimum and maximum for each direction. A negative number indicates that the joint counters the movement of the more proximal joints.

**Table 6:** Angle in degrees of the fingertip path of the right forelimb in lateral view.

Joint contributions to fingertip path angle		
	AF	WAIR
All joints	142.8	101.6
Pitch fixed to 39.6 degrees	125.8	98.4
No wrist	114.0	99.0
No wrist or elbow	119.7	89.0
No wrist elbow or shoulder elevation/depression	54.4	72.3
No wrist elbow or shoulder protraction/retraction	131.0	110.3
No wrist elbow or shoulder pronation/supination	124.0	91.8

## Discussion

Previous investigations of WAIR and of level and descending flight in chukars [4], [5] found that the angle of the wingtip during downstroke falls within a narrow range relative to gravity, despite highly varying orientation of the body. Such movement predicts that shoulder joint motion must be modulated to maintain a relatively constant stroke path relative to gravity. Evidence here from the wing joints demonstrates that the glenohumeral joint controls the vast majority of wing movements (Table 2). More distal joints are primarily involved in modifying wing shape. During both behaviors, all joints are in relatively similar orientations at the top of upstroke and then diverge through downstroke (see Video S2, S3, S4, S5). The primary difference between AF and WAIR glenohumeral movement results from truncated depression of the humerus. Although the obvious explanation is the need for greater sweep of the wing for higher aerodynamic needs during ascending flight, it is also possible that the chukars truncate the wing stroke during WAIR to avoid collisions between the wing and the substrate. There could be a reflex that limits wing excursion when the feet are in contact with the ground.

Ascending flight and WAIR represent opposite ends of the mechanical power spectrum for flapping modes – ascending flight requires much higher mass-specific power [36] to drive the greater aerodynamic forces for wing-only body weight support [8]. Hence the greatly reduced excursion of the wing during WAIR is not surprising. However, considering the fingertip path excursion in the context of these highly varying forces offers interesting insights. Previous studies using external video [4] found that the stroke plane angle in adults was maintained around 120 degrees during WAIR and ca. 135 degrees during both level and descending flight. Here, measuring fingertip in absence of feathers, we found more divergent paths of 102 degrees and 143 degrees. The lower WAIR stroke angle is more consistent with juveniles [4] and may reflect the difference in measuring from feather verses fingertip. However, the similarity of fingertip angle between ascending flight in this study and reported level and descending flight [4] is strongly supportive of a stereotypic wing path.

The steeper path of WAIR is also consistent with angling the aerodynamic force towards the substrate. But what is the underlying cause of the difference in path? It could be the direct result of muscular control (i.e., differential activation of the pectoralis and other muscles). However, it is interesting to consider that the difference in external aerodynamic force could potentially be involved as well. Joint motions result not only from muscular pull but also from the aerodynamic and inertial forces of the wing [37], such that the kinematics are inherently intertwined with the kinetics. Perhaps, the direction of pull during downstroke in WAIR and ascending flight is exactly the same, but consistently less steep during flight behaviors due to variation in “aerodynamic protraction” from the thrust component [37].

### Glenohumeral Joint Motion

Much attention has been focused on understanding control and constraints of the glenohumeral joint [10], [11], [24], [38]–[41]. The most recent interpretation suggests that the acrocoracohumeral ligament (AHL) permits a wide range of glenohumeral paths while simultaneously stabilizing the joint by constraining the combination of rotations and translations [11]. Dorsoventral sliding of the humeral head in the saddle shaped glenoid allows the ligament to maintain tension across a broad array of rotational combinations. We found variation in the translational movement of the humeral head such that the head slides further ventrally during AF. During WAIR, the humeral head rolls in a complex pattern, but is limited to the more dorsal region of the joint surface. During downstroke of AF, the humeral head appears to travel from dorsal to ventral as shown during level flight in starlings (*Sturnus vulgaris*) [40]. The patterns during these two behaviors are consistent with the expected pattern if governed by AHL constraints. However, more detailed tracking of AHL deformation in the future should be done to confirm this hypothesis.

### Furcula and Coracoids

Measures of European starling [23] and magpie (*Pica pica*) [26] furculae showed that the crura spread laterally during downstroke and recoil during upstroke. Chukars also exhibited lateral bending. However, the phasic pattern in flying chukars was reversed, spreading laterally during upstroke and recoiling during downstroke. Additionally, we found AF spreading to be twice that of WAIR (Videos S6, S7). However, this difference in magnitude arose not relative to resting distance, but instead relative to maximum spread. In other words, the furcula expands similarly in both behaviors, but recoils more during AF. These findings should invite further investigations into this matter in order to evaluate the functional significance of these phase-shift observations with respect to furcular bending within a wingbeat cycle.

The ends of the furcula are firmly attached to the coracoids, so bending of the furcula reflects movement of the coracosternal joint. Jenkins et.al [23] investigated several potential causes of coracosternal movement and two particularly enticing hypotheses regarding the role of the furcula arose: 1) an elastic energy storage mechanism, and 2) a secondary respiratory cycling mechanism via compression of the interclavicular air-sacs [42]. However, little further evidence has thus far supported either energy storage [43] or a link between furcular movement and respiration [26], [44].

Several possible mechanisms may explain the pattern seen in chukars. First, resting length is measured on a frozen, dead bird. “Resting” length could be affected by freezing, however, resting length in a thawed chukar compared to frozen showed no difference in interfurcular distance. Hence we interpret this as an accurate resting interfurcular distance. If so, then the coracosternal joint appears to experience a constant abduction moment. Further exploration is needed to assess the cause of this loading pattern, but the difference between AF and WAIR may offer some insight. The greater movement during AF could be due to changing orientation of the resultant forces at the distal ends of the coracoids. The loading on the distal end of the coracoid appears to be dominated by a medial compression of the humeral head on the glenoid and a downward and lateral pull by the acrocoracohumeral ligament, which is placed in tension by the pectoralis or supracoracoideus [10]. Changing relative magnitudes of these components could result in varying torques at the coracosternal joint. Future studies geared specifically towards quantitatively analyzing the interplay of forces responsible for furcular spreading can further take advantage of the wing-loading/kinematic variation of a wider range of flapping behaviors to isolate the cause of interfurcular distance changes found here.

### Elbow and Wrist

All elbow and wrist rotations undergo greater excursion during AF (as with the more proximal joints). These differences likely reflect higher aerodynamic forces during flight, both for generating power during downstroke and reducing drag during upstroke. One notable difference was that the elbow maintains a more abducted posture during AF. This might be expected during downstroke when increased aerodynamic forces would generate a greater abducting torque. However, abduction during AF is retained and, in fact, exaggerated further in upstroke (also to a lesser degree in WAIR).

Many studies have explored the functional and evolutionary implications of the automatic flexion-extension mechanism of birds [35], [45], [46]. In this study, no attempt was made to measure radio-ulnar or carpal movement; rather, we focused on the coarser grained “wrist” movement as the orientation of the metacarpus relative to the ulna. Our results provide two insights that warrant further consideration in studies of wrist movement and mechanics. First, the elbow and wrist flexion timing match closely during WAIR but are offset during AF. During the latter, the extended wrist position is maintained until about 40% of upstroke while the elbow has already fully flexed at this time. The wrist then undergoes rapid flexion during upstroke followed by an extension pattern that is consistent between the two behaviors. Second, the wrist, like the elbow, is capable of abduction/adduction. Perhaps the more complex loading and movement (highly adducted during downstroke) alters the nature of automatic flexion-extension. The pattern of retained extension of the wrist is coincident with timing of wrist adduction.

### Wingbeat Timing

We initially chose the fingertip position to define the turnaround point for upstroke and downstroke. However, we noticed that some joint movements (e.g. humeral elevation/depression) were offset from the upstroke/downstroke transition such that the humerus would begin moving upward prior to the fingertip reaching the bottom of downstroke. This finding led us to consider the possibility of a lag between turn-around timing of different joints.

Many studies of avian wing function rely on downstroke and upstroke transitions to interpret timing of relevant neuromuscular events. EMG [13], [14], [47]–[50], deltopectoral strain [51]–[53], and sonomicrometry [54], [55] are dependent on upstroke/downstroke transition timing for interpreting pectoralis function. Kinematic data from external standard video generally relies on primary tip or wrist position to determine wingbeat timing events (upstroke/downstroke transitions), but the few studies using X-ray define transitions based on the distal humerus [23], [24]. Our results suggest some caution in using the wrist or wing tip to assess of the relative timing neuromuscular events and skeletal timing, particularly during flap-running.

During ascending flight, we did not find evidence of an offset during the upstroke-downstroke transition, but we did find offsets in the downstroke-upstroke transition. This suggests that wrist or wingtip position is in fact reflective of humeral turn-around during the upstroke-downstroke transition but not during downstroke-upstroke. During the latter, the humerus begins moving upward first, followed by the wrist and then followed by the fingertip.

During WAIR, both wing turn-around events showed less clear patterning. Offsets were more variable and larger in magnitude. This may be due to lower wing loadings. The wings are less constrained by aerodynamic requirements. More accurate assessment of this offset should be considered for other flight behaviors and species and higher frame rates are needed to more precisely assess the magnitude.

### Whole Body Movements

Ascending flight was faster than WAIR for traversing the 70 degree incline to an elevated refuge. However, chukars chose to perform WAIR preferentially, only resorting to flight when the ramp was not present. WAIR has been suggested as a predator escape strategy [1], [4] and as a less fatiguing, safer way to ascend to an elevated refuge [36]. WAIR also permits juvenile birds to ascend to refuges prior to having flight capable wings. Familiarity with terrain may also affect the choice of ascent mode. With training on the ramp and learning of the refuge box, our birds readily choose WAIR. However, in the early stages of training they would often burst into flight in multiple directions. It may be that knowing the terrain and refuge location permits them to make a more energetically efficient choice.

It should also be noted that the faster AF speeds limited usable trials because a perfectly timed run was needed to capture a full wingbeat in both camera views. Hence, ascending flight speed may be underestimated since only trials with complete wingbeats were analyzed. Behaviors faster than 2 m/s may not be tenable for the C-arm system for adult chukars if full wingbeats are needed.

### Conclusions

In summary, we present the first look at the skeletal movements during WAIR and ascending flight in adult chukars. Despite substantial differences between individual joint movements, the general path of the fingertip is consistent with the “stereotypic” flight stroke reported from external views, and also consistent with reorienting the aerodynamic force towards the substrate during WAIR. We conclusively show that the glenohumeral joint is the primary influence on distal wing excursion. In terms of individual joints, the humeral head appears to undergo a pattern of movement within the glenoid consistent with the hypothesis that the AHL constrains the movement of the glenohumeral joint. Surprisingly, we found a pattern of furcular spreading opposite that previously reported for starlings and magpies but only during ascending flight, suggesting that the enigmatic underlying cause of furcular spreading may be more complicated than previously thought. Uphill flap-running combined with a broad array of flight behaviors provides a natural experiment in skeletal and joint loading, given the increasing forces associated with the spectrum of behaviors from WAIR at lower to higher angles to descending, level, and ascending flight [36], [50], [56]. This study provides a foundation for broader investigations of skeletal movements during a greater range of behaviors, age classes, and species.

## Supporting Information

Video S1 Rotoscoping method.
(WMV)

Video S2 WAIR (blue) verses AF in a global reference frame.
(WMV)

Video S3 Single trial of each behavior comparing WAIR (blue) verses AF in a sternal reference frame.
(WMV)

Video S4 Eight trials (4 of each behavior) comparing WAIR (blue) verses AF in a sternal reference frame.
(WMV)

Video S5 Average WAIR (blue) verses AF (red) in a sternal reference frame.
(WMV)

Video S6 Furcular spread AF.
(WMV)

Video S7 Furcular spread WAIR.
(WMV)
